# Preliminary Evaluation of a Novel Fetal Guinea Pig Myelomeningocele Model

**DOI:** 10.1155/2021/2180883

**Published:** 2021-08-13

**Authors:** Sarah C. Stokes, Kaeli J. Yamashiro, Melissa A. Vanover, Laura A. Galganski, Jordan E. Jackson, Christina M. Theodorou, Christopher D. Pivetti, Diana Lee Farmer, Aijun Wang

**Affiliations:** ^1^Department of Surgery, University of California-Davis, Sacramento, CA, USA; ^2^Institute for Pediatric Regenerative Medicine (IPRM), Shriners Hospitals for Children Northern California, Sacramento, CA, USA; ^3^Department of Biomedical Engineering, University of California-Davis, Davis, CA, USA

## Abstract

**Introduction:**

Translational models of myelomeningocele (MMC) are needed to test novel *in utero* interventions. An ideal animal model for MMC has locomotor function at birth and is low cost enough to allow for high throughput. The rat MMC model is limited by immature locomotor function at birth. The ovine MMC model is a costly surgical model. Guinea pigs are uniquely suited for an MMC model being a small animal model with locomotor function at birth. We aimed to develop a retinoic acid (RA) model of MMC in the guinea pig and to evaluate if pregnant guinea pigs could tolerate uterine manipulation.

**Methods:**

Time-mated Dunkin Hartley guinea pig dams were dosed with 60 mg/kg of RA between gestation age (GA) 12 and 15 days in the development of an RA model. Fetuses were grossly evaluated for MMC lesions at Cesarean section after GA 31 days. Evaluation of the ability of pregnant guinea pig dams to tolerate uterine surgical intervention was performed by hysterotomy of a separated group of time-mated guinea pigs at GA 45, 50, and 55.

**Results:**

Forty-two pregnant guinea pigs were dosed with RA, with a total of 189 fetuses. The fetal demise rate was 38% (*n* = 71). A total of 118 fetuses were viable, 83% (*n* = 98) were normal fetuses, 8% (*n* = 10) had a neural tube defect, and 8% (*n* = 10) had a hematoma or other anomalies. No fetuses developed an MMC defect. None of the fetuses that underwent hysterotomy survived to term.

**Conclusion:**

RA dosed at 60 mg/kg in guinea pigs between GA 12 and 15 did not result in MMC. Dunkin Hartley guinea pigs did not tolerate a hysterotomy near term in our surgical model. Further work is needed to determine if MMC can be induced in guinea pigs with alternate RA dosing.

## 1. Introduction

Myelomeningocele (MMC) is the most common congenital cause of lifelong paralysis in the United States, and approximately four children are born with this disease daily [1]. The Management of Myelomeningocele Study (MOMS) was groundbreaking in establishing *in utero* surgical repair as the standard of care for MMC and demonstrating potential for reversal of the paralysis associated with the disease [2]. However, while *in utero* MMC repair has improved postnatal outcomes, a significant unmet need remains with only 55% of prenatally repaired children ambulating independently at 30 months, decreasing to 29% at school-age [2–4]. Therefore, there remains a need for ongoing research to develop novel therapeutic agents which may improve outcomes for these patients.

Developing new treatments requires an animal model for safety and efficacy testing. An ideal animal MMC model has locomotor function at birth, tolerates fetal surgical intervention, and has a sufficiently long gestation period to allow for accrual of spinal cord damage and subsequent *in utero* treatment. The ideal animal model must tolerate fetal surgical intervention as these animals will be used to evaluate augmentation of the *in utero* repair of MMC. The gold standard large animal ovine MMC model currently used to study novel fetal MMC surgical interventions fulfills many of these needs: a similar progression of spinal cord damage to humans, a long gestational period, uterine tolerance of multiple fetal interventions, and well-developed fetuses with locomotor function at birth [5]. However, sheep are costly, and the surgically created MMC lesion introduces variability in outcomes from the surgical procedure itself [6, 7]. As a result, studies can be limited by low throughput. Other commonly used models to study fetal MMC interventions are retinoic acid- (RA-) induced MMC lesions in rats and surgically induced MMC lesions in rabbits [8–11]. While these small animal models are less costly than sheep, they are limited by short gestational periods and locomotor immaturity at birth.

Guinea pigs may be uniquely suited for a fetal MMC model. They are less costly than sheep and have a gestation period of 68 days, approximately three times longer than that of rats and two times longer than that of rabbits. This longer gestational period allows enough time for accrual of spinal cord damage and subsequent *in utero* treatment. Furthermore, similar to newborn lambs, guinea pigs have locomotor function at birth. There is evidence that neural tube defects can be induced in fetal guinea pigs with hyperthermia; however, the type of neural tube defect induced is inconsistent [12].

The objective of this study was to develop a RA model of MMC in fetal guinea pigs and to evaluate the ability of guinea pigs to tolerate fetal surgical intervention. We hypothesized that RA would induce MMC in the fetal guinea pig at the same weight-based dose used in rats when given at the same timepoint in gestation as the hyperthermia-induced neural tube defects in guinea pigs. Additionally, we hypothesized that guinea pigs would tolerate uterine manipulation at a single timepoint late in gestation.

## 2. Materials and Methods

Animal work was approved by the Institutional Animal Care and Use Committee, and care was compliant with the *Guide for the Care and Use of Laboratory Animals* (#19877, approved March 15, 2018). The facilities used to conduct this study were accredited by the Association for the Assessment and Accreditation of Laboratory Animal Care, International.

### 2.1. Animal Preparation

Time-mated Dunkin Hartley guinea pigs were used for this study. The day of breeding was designated as gestational age (GA) 1.

### 2.2. Retinoic Acid (RA) Exposure and Evaluation for MMC

All-trans RA (Sigma-Aldrich Chemical, St. Louis, MO, USA) was dissolved in olive oil at room temperature at a concentration of 25 mg/ml, similar to the previously described rat MMC retinoic acid model [9]. All animals in the study were administered RA. Dams were gavage fed the solution within an hour of preparation and were administered a single dose of 60 mg/kg. RA was administered at timepoints between GA 12 and GA 15. Three to four guinea pigs were dosed at each timepoint (Table 1).

As part of model development, four guinea pigs underwent Cesarean section at GA 24 to allow for early identification of MMC defects and inform dose scheduling adjustments. After evaluation of these pups, it was determined that delaying Cesarean section until later in gestation would allow for better evaluation of any anomalies. These 4 guinea pig dams and their pups were excluded from further analysis. Subsequent guinea pig fetuses were evaluated by Cesarean section after GA 31.

All of the fetal evaluations were performed grossly given that the MMC defect is immediately apparent on gross evaluation in the rat model [13], and gross evaluation was successfully used for the determination of development of fetal guinea pig neural tube defects in a hyperthermia model [12]. All defects identified were recorded. Neural tube defects included exencephaly, anencephaly, encephalocele, and myelomeningocele. Guinea pigs that were not found to be pregnant at the time of Cesarean section were excluded from the study.

### 2.3. Evaluation for Tolerance of Uterine Surgical Intervention

**Figure 1:**
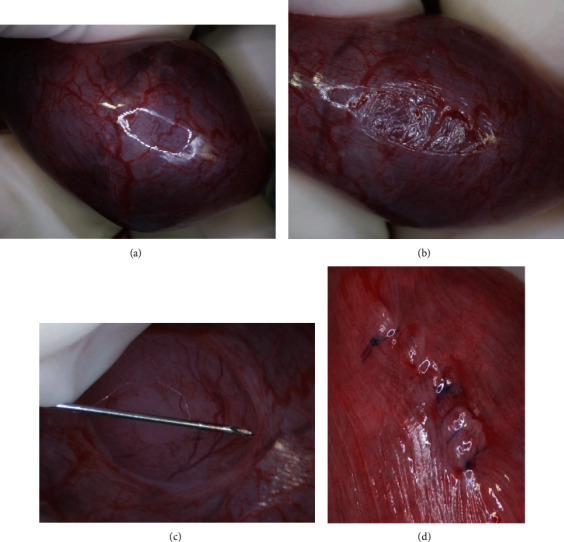
The uterus was exposed (a), followed by a hysterotomy (b), opening of the amniotic sac (c), and repair of the hysterotomy (d).

The ability of pregnant guinea pigs to tolerate uterine surgical intervention was separately evaluated in six guinea pig dams. Guinea pigs that were not previously exposed to RA underwent creation and repair of a hysterotomy at GA 45 (*n* = 3), 50 (*n* = 2), and 55 (*n* = 1) to determine if any uterine surgical manipulation would be tolerated. General anesthesia was induced in an induction chamber with 5% inhalational isoflurane until there was no response to noxious stimuli. The guinea pigs were administered 1 mg/kg of ceftiofur intramuscularly prior to incision. Fur on the abdomen was clipped, and the abdomen was cleaned with 3 rounds of betadine and 70% ethanol. Anesthesia was maintained with 1-5% isoflurane thereafter administered via a facial mask. A midline laparotomy was performed, and the uterus was exposed. The number of pups in each uterine horn was recorded. The majority of the uterus was then returned to the abdomen, leaving only one fetus outside of the abdomen. The fetus was palpated in the uterus, and the uterine wall overlying the fetus was opened with scissors, and an 18-gauge hollow needle was used to open the amniotic sac. Lost amniotic fluid was replaced with warmed normal saline. The amniotic sac and the uterus were closed in a single running layer. The laparotomy incision was closed. Photos of hysterotomy are illustrated in Figure 1.

At GA 62-68, a Cesarean delivery was performed to recover the fetuses and avoid cannibalism by the guinea pig. General anesthesia was induced and maintained using isoflurane. A midline laparotomy was made, and the uterus was exposed. Each uterine horn was opened using scissors. Numbers of living and dead pups were recorded. Pups that were alive were removed from the uterus, and the umbilical cords were ligated. Motor function of pups that survived was video recorded to establish a baseline control for comparison with potential future pups with MMC. They were euthanized at 48 hours.

### 2.4. Data Analysis

The following data were obtained at each surgical intervention: number of fetuses, number of demised fetuses, number of normal fetuses, number of fetuses with gross cranial abnormalities, and number of fetuses with gross caudal abnormalities. As the number of pregnant guinea pigs and fetuses varied by timepoint, the above data were converted to a percentage of the total number of fetuses. Descriptive statistics were performed.

## 3. Results

### 3.1. Ability of Retinoic Acid to Induce Myelomeningocele

**Figure 2:**
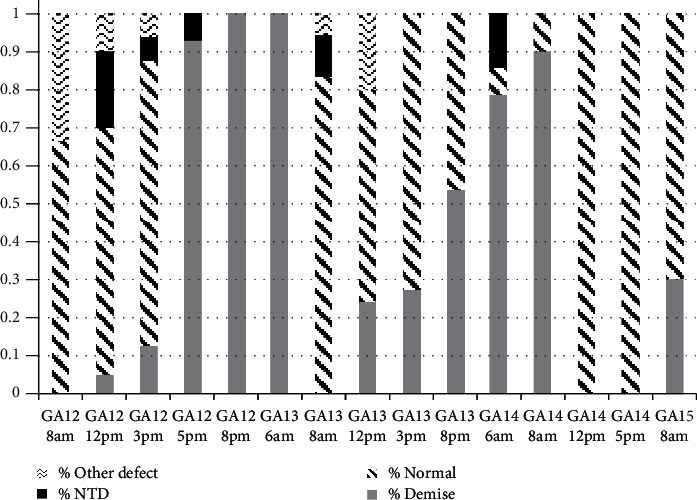
Fetal outcomes of retinoic acid dosing. GA = gestational age.

**Figure 3:**
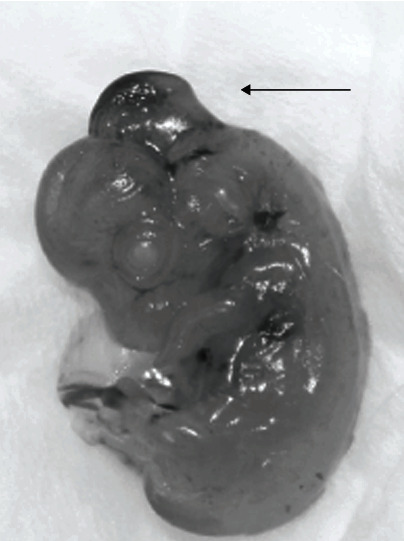
Cranial neural tube anomaly in guinea pig fetus.

Three to four guinea pigs were dosed at each selected timepoint, with a total of 61 pregnant guinea pigs dosed with RA. At the time of Cesarean section, 19 guinea pigs (31% of total dosed) did not have any identifiable fetuses. A total of 42 pregnant guinea pigs were used for evaluation of the effect of RA administration. The median number of fetuses per pregnant guinea pig was 4 (IQR 3-5). There were 189 fetal guinea pigs in total, of which 118 were viable (62%) and 71 demised *in utero* (38%) (Table 1 and Figure 2). On gross evaluation of the viable fetuses, 98 were normal (83%), 10 had neural tube defects (8%), and 10 had other abnormalities (8%). All of the neural tube defects were cranial defects. An image of a guinea pig pup with a cranial neural tube defect is illustrated in Figure 3. No fetuses developed MMC defects. The other abnormalities included hematoma around the skull (*n* = 1), hematoma at the skull base (*n* = 1), cervical hematoma (*n* = 1), thoracic hematoma (*n* = 3), lumbar hematoma (*n* = 3), and hydrops (*n* = 1).

Cranial neural tube defects occurred at the highest rate when dams were dosed at GA 12 at 12pm (20%) (Table 1 and Figure 2). Cranial neural defects were also observed at timepoints immediately following this, at GA 12 at 3pm (6%) and at GA 12 at 5pm (7%). Cranial neural tube defects were also observed with RA administration at slightly later timepoints, at GA 13 at 8am (11%) and GA 14 at 6am (14%). Rates of fetal demise were highest at GA 12 at 5pm (93%), GA 12 at 8pm (100%), GA 13 at 6am (100%), and GA 14 at 8am (90%).

### 3.2. Guinea Pig Tolerance of Hysterotomy

A hysterotomy was performed on six additional pregnant guinea pigs which did not receive RA at GA 45 (*n* = 3), GA 50 (*n* = 2), and GA 55 (*n* = 1) shown in Table 2. Images of the hysterotomy creation and closure are provided in Figure 2. Four (4/6, 67%) guinea pig dams did not survive to planned Cesarean section at GA 62-64. Of the three dams who underwent hysterotomy at GA 45, two demised prior to planned Cesarean section. One was found dead on postoperative day two; on necropsy, she was found to have blood in her uterus. One became lethargic and anorexic on postoperative day two; ultrasound was performed on postoperative day 6, and all fetuses were found to be dead; she was euthanized. Neither of the dams who underwent hysterotomy on GA 50 survived to Cesarean section. One became lethargic and developed teeth chattering on postoperative day two and was euthanized. At necropsy, all pups were identified to have demised prior to euthanasia. The other dam aborted two of her fetuses on postoperative day one and two more on postoperative day two. No further fetuses were identified on ultrasound, and she was euthanized.

Of the two surviving guinea pigs, one had undergone hysterotomy at GA 45 and the other at GA 55. At the time of Cesarean section, both of the fetuses over which the hysterotomy was performed had demised. In the dam in which hysterotomy was performed at GA 45, two of the other pups had demised and two survived. In the dam in which hysterotomy was performed at GA 55, the three other pups survived. The hysterotomy site was well healed.

## 4. Discussion/Conclusion

This pilot study sought to develop a RA-induced MMC model in the fetal guinea pig and to evaluate tolerance of hysterotomy in guinea pigs. Administering a single dose of all-trans RA at 60 mg/kg between GA 12 and 15 did not result in any MMC defects. No fetal guinea pig at the site of a maternal hysterotomy survived to term. While the guinea pig is theoretically a uniquely suited model to study fetal MMC interventions, the RA-induced MMC model was unsuccessful in this pilot study. Furthermore, our results suggest that guinea pigs may not tolerate uterine surgical intervention, which would impede their potential use as a model for *in utero* surgical MMC repair.

We chose to administer a single dose of all-trans RA at 60 mg/kg based on studies performed in mice, hamsters, and rats. Dose range finding studies have demonstrated an optimal dose of 60 mg/kg in rats and hamsters [9, 14]. Higher doses result in the death of multiple fetuses, with a dose of 80 mg/kg resulting in 37% fetal demise in rats [15]. If the administered dose is too low, it can result in fetal demise when administered early in gestation, and no defects when administered just prior to neural tube closure. In hamsters, if a low dose, 20 mg/kg, is administered before GA 7.5, 50% of pups die; when 20 mg/kg is administered at GA 8.75, the appropriate time for MMC induction with a higher dose, there are no MMC defects [16]. Administering a single dose to each guinea pig rather than multiple doses was chosen as administering RA multiple times to the same mouse during the critical window has been demonstrated to result in high rates of resorption and fetal death [17]. A potential explanation for the failure of this dose in guinea pigs is variation in all-trans RA metabolism between species. While guinea pig metabolism of RA has not been evaluated, pregnant rats and rabbits have been demonstrated to metabolize RA differently [18]. Potential future directions include attempting an increased dose of RA or measuring the placental concentration of RA after administration to guide optimal dosing.

Timing of administration of RA was based on studies performed in other rodent models and knowledge of guinea pig fetal development. In hamster, mouse, and rat models, administration of RA just prior to neural tube closure has resulted in MMC neural tube defects [9, 16, 19]. The timing of neural tube closure varies between rodents. In guinea pigs, neural tube closure has been reported between GA 14 and 18 [20]. Prior models have demonstrated that inducing hyperthermia in a guinea pig on GA 12-14 results in neural tube defects [12, 21]. In hamster and mouse models, timing of hyperthermia to induce neural tube defects has been the same as timing of RA to induce neural tube defects [22, 23]. Therefore, we studied timepoints between GA 12 and GA 15 as this was just prior to the timing of neural tube closure in a guinea pig, and this window was successful in hyperthermic induction of neural tube defects. When guinea pigs were heated, neural tube defects were induced in 12% with double heating on GA 13, 18% with double heating on GA 14, and 10% with single heating on GA 13 [12]. Notably, in this prior study, though a total of 22 guinea pig pups had neural tube defects induced with hyperthermia, only one of these defects was caudal [12]. There are a number of potential reasons for our lack of success in creating MMC defects with this timing. The window in other rodents for RA administration is very narrow with some reports of only hours, and it is possible that we missed the critical window or the window for guinea pigs is outside of the range we evaluated. In other rodents, if RA is administered prior to the critical window, there is a high rate of fetal demise and exencephaly [9]. If RA is administered after neural tube closure, animals will have defects such as limb malformations but will not develop MMC [16]. In our study, the highest rates of fetal demise occurred between GA 12 at 5pm–GA 13 at 6am and GA 13 at 8pm–GA 14 at 8am. From GA 14 at noon onwards, the majority of fetuses survived and were normal. It is also possible that we dosed the guinea pigs with too low a dose, as a lower dose early in gestation could have resulted in death on GA 12-13, while not causing MMC in later gestation pups.

There are multiple areas to continue to explore the development of a guinea pig MMC model. These results provide preliminary data to form the basis of these future investigations on optimal dose timing and optimizing maternal and fetal survival. In presenting these results, we aim to uphold the principle of reduction in animal research and ensure that other groups will not unnecessarily replicate our attempts. There is tremendous value in establishing a high-throughput small animal MMC model with locomotor function at birth.

In order to function effectively as a model for *in utero* intervention for MMC, an animal model must be able to tolerate fetal surgery. Ultimately, in a guinea pig model, we would induce MMC with RA, allow time for accrual of spinal cord damage, and then perform *in utero* surgical intervention. We therefore chose timepoints late in gestation so there would be sufficient time to allow for accrual of spinal cord damage after RA administration and prior to repair. The guinea pig has been used for other fetal models evaluating fetal wound healing and fetal cardiac development [24, 25]. In the fetal wound healing model, 144 fetuses of 74 guinea pigs (breed not specified) underwent general anesthesia with intervention at mid to late gestation. In all cases, the guinea pig dam died or the fetuses were aborted [24]. However, in the fetal cardiac model, 6 mixed-breed guinea pigs underwent spinal anesthesia at GA 50-52 with a 50% fetal mortality rate and a 5% maternal mortality rate [25]. Surgical intervention with general anesthesia in nonpregnant guinea pigs has been reported to have marginally better survival rates compared to rats, with 63% of guinea pigs alive at 24 hours compared to 44% of rats in an aortic graft model [26]. The 100% mortality rate of the fetuses in dams who underwent hysterotomy in our study and that of the fetal wound healing model may be due to the use of general anesthesia. The maternal mortality rate of 67% is likely related to the high rate of fetal demise, with dams in our study appearing to develop systemic illness rather than abort the fetus. Another possible explanation is that the mixed-breed guinea pig may be able to tolerate fetal surgical intervention whereas the Dunkin Hartley guinea pigs may not.

There are several limitations of this study. Only a single dosage of RA was used across all timepoints. This dose was chosen based on the efficacy of 60 mg/kg in the induction of MMC defects in rats and hamsters [9, 14]. Investigation into the potential for different doses to induce MMC in the hamster is an area for further investigation. It is possible that multiple doses may be needed to induce MMC. Additionally, each timepoint had varying pregnancy rates and numbers of fetuses per guinea pig. It is possible that guinea pig dams found to be not pregnant at the time of Cesarean section had fetal demise and resorption induced by RA. However, even when RA is administered multiple times to a single pregnant rat very early in gestation, the rate of all fetuses in a pregnancy resorbing in rat is only 29% [27]. When RA is administered multiple times to rats between GA 12 and 16, only 7% of rats had resorption of all fetuses [27], and we therefore suspect that the majority of these guinea pigs were not pregnant at the time of RA administration. All fetal evaluations were performed grossly, and therefore, some of the abnormalities could be inappropriately characterized. However, histologic analysis of overtly normal-appearing RA exposed fetal rats has not demonstrated any spinal defects [9]. Additionally, we did not evaluate a control group and were unable to perform any statistical evaluation. A relatively small sample size of guinea pigs was evaluated for tolerance of hysterotomy, and potentially with increased researcher experience, this surgery would be survivable for the guinea pig dams and fetuses.

In conclusion, RA dosed at 60 mg/kg in guinea pigs between GA 12 and 15 does not result in MMC. Dunkin Hartley guinea pigs did not tolerate fetal surgical intervention under general anesthesia. These results provide data to guide future development of RA-induced MMC models in the guinea pig.

## Figures and Tables

**Table 1:** Fetal outcomes at retinoic acid dosing times. GA = gestational age in days.

		Total number of guinea pigs dosed	Pregnant guinea pigs dosed	Total fetuses	Viable	Demise, *n* (% of total fetuses)	Normal, *n* (% of total fetuses)	NTD, *n* (% of total fetuses)	Other defects, *n* (% of total fetuses)
GA12	8am	4	1	3	3	0 (0)	2 (67)	0 (0)	1 (33)
	12pm	4	4	20	19	1 (5)	13 (65)	4 (20)	2 (10)
	3pm	4	3	16	14	2 (13)	12 (75)	1 (6)	1 (6)
	5pm	4	2	14	1	13 (93)	0 (0)	1 (7)	0 (0)
	8pm	4	3	15	0	15 (100)	0 (0)	0 (0)	0 (0)

GA13	6am	4	1	1	0	1 (100)	0 (0)	0 (0)	0 (0)
	8am	4	4	18	18	0 (0)	15 (83)	2 (11)	1 (6)
	12pm	4	4	25	19	6 (24)	14 (56)	0 (0)	5 (20)
	3pm	4	3	11	8	3 (27)	8 (73)	0 (0)	0 (0)
	8pm	4	4	13	6	7 (54)	6 (46)	0 (0)	0 (0)

GA14	6am	4	3	14	3	11 (79)	1 (7)	2 (14)	0 (0)
	8am	4	3	10	1	9 (90)	1 (10)	0 (0)	0 (0)
	12pm	3	2	8	8	0 (0)	8 (100)	0 (0)	0 (0)
	5pm	3	3	11	11	0 (0)	11 (100)	0 (0)	0 (0)

GA15	8am	3	2	10	7	3 (30)	7 (100)	0 (0)	0 (0)

**Table 2:** Surgical results. Six dams underwent hysterotomy. GA = gestational age in days.

GA	Fetal outcome	Maternal outcome
45	Demise	Survived
45	—	Expired
45	—	Failure to thrive and euthanized
50	—	Failure to thrive and euthanized
50	Aborted	Euthanized after aborted fetuses
55	Demise	Survived

## Data Availability

Data is available on request.
